# Nanoindentation analysis of the micromechanical anisotropy in mouse cortical bone

**DOI:** 10.1098/rsos.160971

**Published:** 2017-02-22

**Authors:** Michele Casanova, Anna Balmelli, Davide Carnelli, Diana Courty, Philipp Schneider, Ralph Müller

**Affiliations:** 1 Institute for Biomechanics, ETH Zürich, Zürich, Switzerland; 2 Complex Materials, Department of Materials, ETH Zürich, Zürich, Switzerland; 3 Laboratory for Nanometallurgy, Department of Materials, ETH Zürich, Zürich, Switzerland; 4 Bioengineering Science Research Group, Faculty of Engineering and the Environment, University of Southampton, Southampton, UK

**Keywords:** nanoindentation, mouse cortical bone, mechanical anisotropy, reduced modulus, hardness

## Abstract

Studies investigating micromechanical properties in mouse cortical bone often solely focus on the mechanical behaviour along the long axis of the bone. Therefore, data on the anisotropy of mouse cortical bone is scarce. The aim of this study is the first-time evaluation of the anisotropy ratio between the longitudinal and transverse directions of reduced modulus and hardness in mouse femurs by using the nanoindentation technique. For this purpose, nine 22-week-old mice (C57BL/6) were sacrificed and all femurs extracted. A total of 648 indentations were performed with a Berkovich tip in the proximal (P), central (C) and distal (D) regions of the femoral shaft in the longitudinal and transverse directions. Higher values for reduced modulus are obtained for indentations in the longitudinal direction, with anisotropy ratios of 1.72 ± 0.40 (P), 1.75 ± 0.69 (C) and 1.34 ± 0.30 (D). Hardness is also higher in the longitudinal direction, with anisotropic ratios of 1.35 ± 0.27 (P), 1.35 ± 0.47 (C) and 1.17 ± 0.19 (D). We observed a significant anisotropy in the micromechanical properties of the mouse femur, but the correlation for reduced modulus and hardness between the two directions is low (*r*^2^ < 0.3) and not significant. Therefore, we highly recommend performing independent indentation testing in both the longitudinal and transverse directions when knowledge of the tissue mechanical behaviour along multiple directions is required.

## 1. Introduction

Bone has a particular hierarchical structure and it is recognized that changes occurring at lower hierarchical levels can affect the functionalities of the whole bone [1,2]. Many insights into the biology and biomechanics of bone tissue at multiple hierarchical levels have emerged from animal experiments. Rodent models are of prime importance as they are inexpensive, easy to breed and a relatively high number of animals can be bred concurrently [3]. Moreover, inbred rodents have negligible genetic variation, which drastically reduces biological variance [3]. Mouse models, in particular, can be used for gene targeting technologies and antibody-mediated suppression of protein functions [3], which are crucial for investigating the genetic fingerprint of bone cells expression. Despite the fact that rodents have become the preferred system for bone research [4], there is still a lack of knowledge on the mechanical behaviour of mouse bone. A deeper understanding of the mechanics in different directions is required to better comprehend the effect of any treatments on the bone tissue.

In recent years, nanoindentation has emerged as a powerful technique for investigating the micromechanical properties of bone [5]. In nanoindentation measurements, a tip penetrates the material while the reaction forces and the depth of penetration are recorded. From this data, parameters related to the stiffness and strength of the indented region can be determined [5]. This technique allows the decoupling at the microscopic scale of the mechanical properties in multiple directions. In particular, the transverse direction may be strongly correlated to bone strength. It is known that most fractures in long bones are rarely owing to mere flexion, but also owing to compressive and torsional forces [6,7]. Moreover, torsion of long bones generates circumferentially oriented shear stresses inside the structure. These stresses can consequently create longitudinal microcracks in the osteons, which can contribute to fatigue failure in cortical bone [6]. It was already observed that shear stresses can induce microcracks generated by shear displacements in bovine cortical bone [8]. Although mouse cortical bone does not present an osteonal structure, microcracks have also been shown to occur in rodents [8,9], and to form preferentially along longitudinal canals [10]. Furthermore, the femoral numerical crack density in rats was found to be considerably greater than in the bovine tibia [8].

Despite the fact that there are many studies on human or bovine cortical bone focused on the mechanical properties in both longitudinal and transverse directions [11–21], investigations into the mouse bone transversal direction have been fewer in number [22–24]. However, a deeper comprehension of bone anisotropy could help understand the basic mechanical properties of mouse cortical bone.

The aim of this study is to shed light on the micromechanical properties along the longitudinal and transverse directions in the mouse femoral shaft and to determine a relationship between them. We expect to find significant differences between properties in these directions because of the anisotropic organization of the bone matrix. To understand the structure–property relationship of mouse bone, micromechanical properties were measured on the same mice, as it is well known that micromechanical properties between two different animals can greatly differ [5]. In particular, three regions of the femoral shaft of mice with completely matured cortical bone were selected and the reduced modulus and hardness were measured by arrays of indentations. Our findings could help to design future studies, because if a constant anisotropy ratio is found, the properties in the orthogonal direction could possibly be inferred from data in a single direction only.

## 2. Material and methods

### 2.1. Specimen preparation

**Figure 1.**
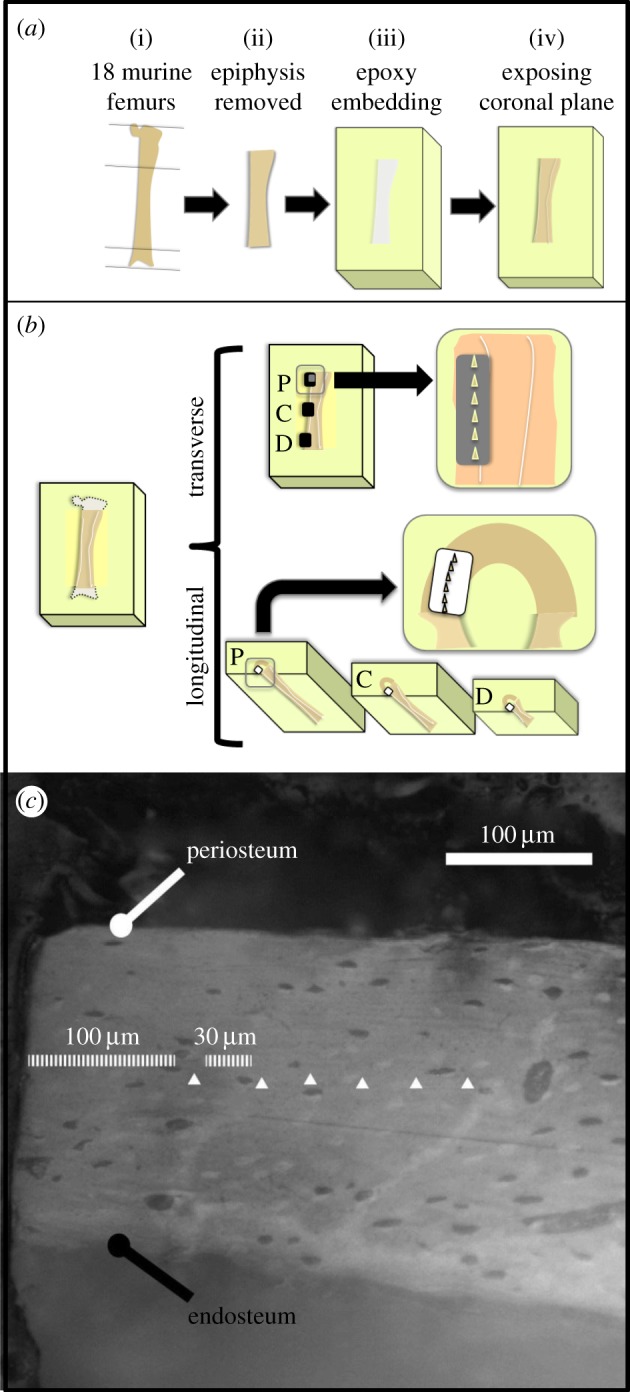
(*a*) Schematic of the sample preparation procedure. (*b*) Location of the indentations on the mouse femur in the proximal (P) central (C) and distal (D) regions. (*c*) Image reporting the location of the indentations in the transverse direction (white triangles). Visible lacunae were carefully avoided.

Nine C57BL/B6 mice were sacrificed with cervical dislocation at the age of 22 weeks and immediately stored in a freezer at −20°C. All animal procedures were approved by the local veterinary authorities (Kantonales Vetrinaeramt Zürich, Zürich, Switzerland). The mice were subsequently thawed and both femurs extracted (figure 1*a*(i)). The proximal part of the femur was disconnected at the end of the third trochanter and the distal part was removed at the end of the condyle with a wire saw (WELL Diamond Wire Saw, LeLocle, Switzerland) to facilitate handling of the femoral shaft (figure 1*a*(ii)). To cool down the wire during the cut, the wire saw liquid tank was filled with phosphate buffered saline (PBS) solution. Epoxy resin (EpoxiCure, Buehler, Lake Bluff, IL, USA) was then used to embed the shafts (figure 1*a*(iii)). This embedding medium was selected to avoid infiltration of the polymer in the bone porosity, which is greatly limited owing to its high viscosity and fast solidification. The bones were then cut longitudinally in order to expose their posterior part, 100 µm before the central coronal plane. The surfaces were polished using increasing grades of carbide papers (P1200, P2500 and P4000), using abundant quantities of PBS as a cooling agent. An alumina solution of grain size 50 nm mixed with PBS was used for the final polishing. After this procedure, the central coronal plane was exposed (figure 1*a*(iv)). Finally, an ultrasonic bath with PBS was performed for 120 s to remove all residues.

After testing along the transverse direction (figure 1*b*), samples were ground and polished to expose femur cross sections. To ensure the perpendicularity of the surface to the bone long axis, a special custom-made holder was used during grinding and polishing procedures. Carbide papers P320 and P500 were used to remove material. When approaching the surface of interest, carbide paper P1200, P2500, P4000 and alumina solution were used for the final polishing as explained above. Three femoral shaft cross sections per sample corresponding to the proximal, central and distal positions along the long axis of the bone were exposed for testing in the longitudinal direction (figure 1*b*). These three testing locations were selected in order to have distinct regions on the femur shaft, which were as far apart as possible but still on the cylindrical portion of the shaft. All samples were then washed in an ultrasonic bath as previously described.

Finally, samples were wrapped in PBS-soaked gauze, snap-frozen and stored at −20°C. Directly before testing, samples were thawed and subsequently immersed in PBS at room temperature for 30 min to assure hydration.

### 2.2. Nanoindentation tests

This subchapter starts by describing the characteristics of the indentations we performed. The locations of the indents are subsequently explained in §2.2.1 and 2.2.2. Before testing, the topography on regions of at least 0.01 mm^2^ of all polished surfaces was measured with an optical profilometer (PLu neox, Sensofar-Tech, Terrassa, Spain). Linear profilometries with a total length of 2 mm were traced on these surfaces, avoiding canals and lacunae present on the lines. The profilometries were analysed with SensoMap 6.1 (Sensofar-Tech, Terrassa, Spain). The average sample surface roughness (Ra) was controlled in the region to be indented and only surfaces with roughness of less than 0.05 µm were accepted for indentation [25].

Nanoindentation tests on the femoral shafts were performed with a TI 900 Triboindenter (Hysitron Inc., Minneapolis, MN, USA) with a Berkovich tip. A fused silica reference sample was used to calibrate the tip area function and machine compliance by performing 100 indentations between 100 and 10 000 µN maximum force [26]. A ramp-and-hold protocol with a maximal load of 6000 µN was applied. A loading rate of 300 µN s^−1^, a holding time of 30 s at maximal load and an unloading rate of 900 µN s^−1^ were chosen to perform the measurements. The 30 s holding time was adopted to eliminate creep effects [27]. Sets of six indentations each were performed for the three regions in each sample. At the beginning of each set, an optical calibration was performed on an aluminium reference sample to ensure the correct positioning of the tip on the sample. All indentations were located in the cortical bone at equal distance from the periosteum and the endosteum (figure 1*c*). A 100 µm distance from the edge of the orthogonal surface was taken for avoiding regions where grinding or cutting might have generated microfractures in the bone. Rows of indentations with 30 µm spacing were taken at each site. Since the area of imprint was approximately 10 µm^3^, which corresponds to a contact diameter of about 7 µm, no overlap between indents occurred [21]. A total of 648 indentations were performed.

The Oliver–Pharr method [28] was applied to evaluate the reduced modulus and hardness of the tissue from the unloading branch of the load–depth indentation curve. This method assumes that the unloading part of the load–displacement graph is linear elastic, which explains the elastic contact stiffness (*S*) and the reduced elastic modulus (*E*_r_) as follows:

2.1 $$E_{r}=\frac{1}{\beta }\frac{S\sqrt{\pi }}{2\sqrt{A}}\;,$$

where *β* is the geometrical parameter and *A* is the contact area. The value for *S* was evaluated by fitting the unloading segment from 95% to 40% of the maximum load. The hardness (*H*) can be found as the maximum load (*P*_max_) divided by the contact area (*A*):

2.2 $$H=\frac{P_{\max}}{A}.$$

#### 2.2.1. Experiments in the transverse direction

The proximal indentations in the transverse direction were located distally from the third trochanter of the femurs on their lateral side. This corresponds to 45% of the whole femoral length. The central indentations in the longitudinal direction were performed at 65% of the total femoral length, whereas the distal indentations were performed at 80% of the whole femoral length (figure 1*b*). Before every set of six indentations, the sample was re-immersed in PBS for 5 min for rehydration and the surface was wiped with Kimtech tissue paper (Kimberly-Clark, Irving, TX, USA) to remove excessive water.

#### 2.2.2. Experiments in the longitudinal direction

Indentations in the longitudinal direction were located in accordance with the indentation location in the transverse direction. The cross sections were indented at 45% (proximal), 65% (central) and 80% (distal) of the femoral length, starting proximally. The indentations were performed in a curved line to keep indentations in the central portion of the cortical bone (figure 1*b*).

### 2.3. Statistical analysis

Statistical analysis was performed using R Statistical Software (Foundation for Statistical Computing, Vienna, Austria). Student's *t*-tests were performed between the longitudinal and transverse directions in the three regions of interest (proximal, central and distal locations), on reduced modulus and hardness for both orientations (e.g. reduced moduli of indentations in the transverse direction in a proximal location versus reduced moduli of indentations in the longitudinal direction in a proximal location). A one-way ANOVA with Bonferroni's *post hoc* test was performed between the three regions of interest on reduced modulus of indentations in the same orientation. It was, therefore, performed between the proximal, central and distal locations. The same statistical analysis was also performed for hardness in the mentioned regions. Moreover, a paired sample *t*-test was performed between the results obtained on left femurs and right femurs. Mean reduced moduli and hardness between the respective regions (e.g. reduced moduli in the transverse central region of left versus right femurs) were tested for bilateral differences. For investigating possible correlations between the longitudinal and transverse direction for reduced modulus and hardness, the Pearson product–moment correlation coefficients were computed.

## 3. Results

**Figure 2.**
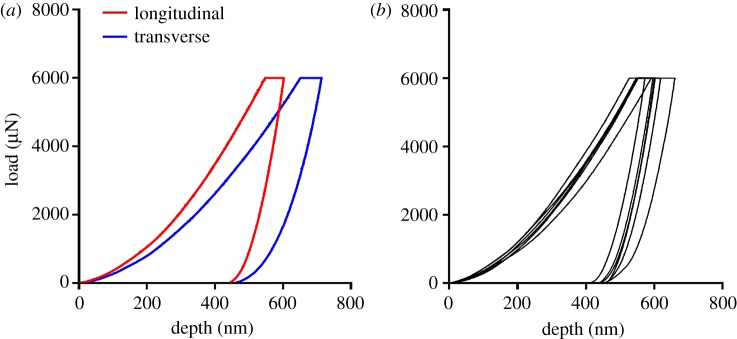
(*a*) Representative indentation curves along the longitudinal and transverse directions in the central regions of sample M1 L (mouse 1, left leg). (*b*) Load–depth curves obtained for the six indents along the longitudinal direction in the proximal region of sample M1 L.

Figure 2 depicts examples of load–displacement curves obtained in this study. Figure 2*a* shows representative curves for longitudinal (red curve) and transverse (blue curve) directions in the central region of sample M1 L (mouse 1, left leg). A difference in penetration depth is evident. Figure 2*b* presents load–displacement curves obtained for the six indents along the longitudinal direction in the proximal region of sample M1 L. Indents within the same region tend to have similar penetration depth.

Two set of indentations were discarded owing to a misplacement of the indents (transverse indentation of samples M4 L central and M5 L distal). Mean results for reduced modulus of the set of six indents for the transverse and longitudinal directions range between a minimum mean of 6.75 ± 0.50 GPa (sample M2R, transverse direction, proximal region) and a maximum mean of 23.81 ± 2.47 GPa (sample M5R, longitudinal direction, proximal region). Mean hardness for the set of six indents along transverse and longitudinal directions range between a minimum of 0.38 ± 0.068 GPa (sample M5R, transverse direction, central region) and a maximum of 0.82 ± 0.092 GPa (sample M6R longitudinal, proximal region). The results of reduced modulus and hardness for each set of indentations in the form of bar charts can be found in the electronic supplementary material, figure S.1.

**Figure 3.**
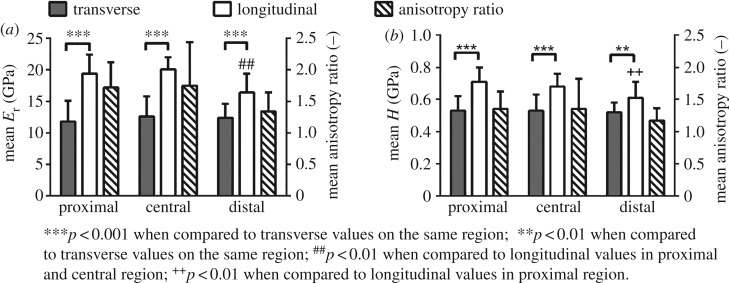
Bar charts showing the mean value and standard deviation of reduced modulus (*E*_r_, *a*) and hardness (*H*, *b*) in the longitudinal and transverse directions in the three analysed regions. Mean anisotropy ratio for each measurement is also reported.

The mean values and standard deviations of reduced modulus and hardness for the three analysed regions (proximal, central and distal) are reported in figure 3 (numerical values provided in the electronic supplementary material, table S.1). The anisotropy ratio is defined as the ratio between the longitudinal and transverse values of the mechanical property of interest. Mean anisotropy ratios for both measurements are also shown in figure 3. Anisotropy ratios for the reduced modulus are of 1.72 ± 0.40 (proximal), 1.75 ± 0.69 (central) and 1.34 ± 0.30 (distal), whereas ratios for the hardness are of 1.35 ± 0.27 (proximal), 1.35 ± 0.47 (central) and 1.17 ± 0.19 (distal). We observed significant differences between the longitudinal and the transverse direction for both reduced modulus and hardness. *p*-value is lower than 0.001 in all cases except for the hardness between the two directions in the distal position (*p* < 0.01). Moreover, significantly lower values of reduced modulus in the longitudinal direction are detected in the distal region as compared to the proximal and central regions (*p* < 0.01), whereas for hardness in the longitudinal direction only the proximal region significantly differs from the distal region (*p* < 0.01). No statistical difference was observed in the mean reduced modulus and hardness between the results from the left and right femurs in the transverse and longitudinal directions.

**Figure 4.**
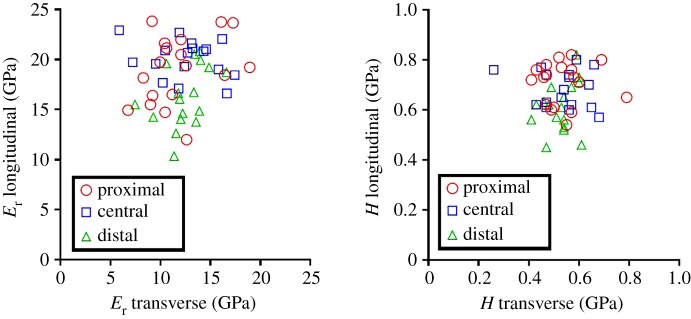
Scatter plot for the reduced modulus (*E*_r_, *a*) and hardness (*H*, *b*) found in the two orthogonal directions in the proximal, central and distal locations. Each element in the plot represents a specific location (proximal, central or distal) of the same femur (18 femurs in total). Two points were removed (central and distal regions) owing to a misplacement of two set of indentations. No correlation was found.

A scatter plot of the reduced modulus and hardness values in the two orthogonal directions is depicted in figure 4. No correlation is observed between longitudinal and transverse directions for either reduced modulus or hardness.

Reduced modulus and hardness for each single indentation can be found in the electronic supplementary material in .xls and .sav format.

## 4. Discussion

This study aims at the investigation of the anisotropic mechanical properties of the mouse femur by performing indentation tests in transverse and longitudinal directions in three locations of the femoral shaft. The mechanical properties of mouse cortical bone were already evaluated at the microscale in various studies [29–32]. The values of reduced modulus and hardness found in this work are lower compared to those of the literature data on mouse bone. Differences in the indentation protocol and method used for data analysis can influence the results [5]. Nevertheless, the main factor leading to these higher values is tissue dehydration. In our study, experiments were performed on wet bone, whereas most studies are performed on dry tissue, which causes an increase of both reduced modulus and hardness by 20–30% [33]. This holds true also for studies on human or bovine bone [33–35]. Other studies with experiments performed in a wet environment and along orthogonal directions found results in line with our data [36–38].

Large animals present structures which encompass many hierarchical levels and make a comparison with the simpler circumferential lamellar structure of the mouse femur rather difficult. Collagen fibres' orientation within the osteon is believed to be the principal reason for differences in the micromechanical properties in cortical bone along different directions in large animals and humans [20,39–41]. Rodent bone does not present an osteonal structural organization, but their collagen fibres are also mainly oriented in an axial direction in their long bones [42]. Literature studies on human and bovine bone found an anisotropy ratio of around 1.5 for the elastic properties at the microscale [13,14,17,18,43–45]. Hardness was investigated in significantly less studies with anisotropy ratios varying from 1.1 to 1.3 [17,18,44]. The mentioned ratios are similar to what is obtained in our study on mouse femurs (see Results section and figure 3). A difference in the anisotropy ratio between reduced modulus and hardness is also found. While the reduced modulus represents the elastic behaviour of the tissue, the hardness is related to failure mechanisms such as slippage at the collagen–mineral interface [46], phase transformation of the mineral phase [47] and sacrificial bond disruption between fibrils [48] that determine its inelastic deformation. As the hardness of the tissue is less anisotropic than its reduced modulus, there is evidence that these inelastic phenomena contribute to a reduction in the anisotropy in the failure behaviour compared with the elastic behaviour.

The mean anisotropy ratios of the reduced modulus and hardness found in this study (figure 3) are similar in the proximal and central regions. However, lower values are obtained in the distal region. This difference can be attributed to the micromechanical properties in the longitudinal direction, which differ from proximal and central regions. Indeed, the mean value of the distal longitudinal indentations is significantly lower to at least one of the other two groups of longitudinal indentations for both the reduced modulus and hardness (*p* < 0.001). It is challenging to find a reason for the behaviour in this region of the femoral shaft, as differences in mechanical properties compared with the other two regions are not found in the transverse direction. It is possible that the change in geometry in this part of the femoral shaft leads to these lower values, since the distal part of the femur tends to gradually increase its diameter towards the condyle. This could cause the collagen fibres to no longer be parallel to the indentations in the longitudinal direction. On the other hand, transverse indentations could still be perpendicular to the collagen fibres, a fact that would justify the similarity of the results in the proximal and the central region.

In studies focusing on the microscopic properties of bone, a higher variance in modulus and hardness of the indentations in the transverse direction is often observed [13,14,17,18,49]. This is also the case for our experiments, where the relative standard deviation in the transverse direction is always higher compared to the longitudinal direction. This general behaviour could possibly be explained by the structure of the collagen bundle. It is hypothesized that the collagen fibres tend to structure themselves in bundles which might be cemented together owing to non-collagenous proteins [50,51]. Therefore, the larger variability in mechanical responses when indenting perpendicularly to them might be because of the presence of a less heterogeneous structure in the transverse compared with the longitudinal direction owing to the preferred axial directionality of the bundles. However, these remain hypotheses since the existence of bundles is still debated. It is also worth noticing that the higher variation of elastic properties in the transverse direction was observed at the mesoscale in cortical portions of long bones in larger animals [13,19,49,52] and it also seems that the larger variability when indenting perpendicularly is reflected at the whole bone level.

It is known from the literature that a strong correlation exists between the reduced modulus and hardness in the same direction [53]. High correlation was found also in our data (transverse *R*^2^ = 0.81, *p* < 0.01; longitudinal *R*^2^ = 0.68, *p* < 0.01). On the other hand, it is interesting to observe that no correlation was found between the transverse and longitudinal direction within single regions either for the reduced modulus or for hardness (figure 4). Apparently, the micromechanical properties of the bone in the longitudinal direction seem not to be a strong predictor of the properties in the transverse direction and vice versa.

Some limitations of this study warrant discussion. The microscope positioning system was calibrated relative to the nanoindenter before every set of indentations to assure that the indent location was effectively distant from the lacunae. However, it is uncertain whether a lacuna might be positioned under the indentation point and hence jeopardize the results. Our set encompassing six indentations should partially correct this potential bias. Owing to the high number of indentations, multiple freezing and thawing of the samples was necessary. This procedure has the potential to partly alter mechanical properties. However, we paid attention to creating conditions for a rapid transition from 0°C to −10°C, as this is known to be critical for avoiding destructive ice crystals [5]. In this study, we did not perform whole bone mechanical testing (e.g. torsion testing or 3-point bending) owing to the limited amount of samples at our disposal. However, this might have been beneficial in order to better understand the implications of the micromechanical properties at a whole-bone level.

## 5. Conclusion

In this paper, we investigate the anisotropic micromechanical properties of the mouse femur by nanoindentation. The reduced modulus and hardness of femoral mouse cortical bone were measured in three distinct regions both in the longitudinal and transverse directions. Anisotropy ratios were found in proximal, central and distal regions of the mouse femur shaft. However, a clear intrasample correlation between the transverse and longitudinal planes in terms of elastic properties and hardness is missing. This leads to a high standard deviation of the anisotropy ratios in all three of the analysed regions. Therefore, it seems that relevant mechanical properties on orthogonal planes cannot be inferred from measurements in a single direction in individual samples. This finding suggests that the measurement of micromechanical properties in the femoral shaft in multiple directions is necessary in order to obtain a precise phenotyping.

## Supplementary Material

Table S1

## Supplementary Material

Figure S1

## Data accessibility

The datasets supporting this article have been uploaded as part of the supplementary material and on the Dryad Digital Repository: http://dx.doi.org/10.5061/dryad.h5p79 [54].

## References

[1] Weiner S, Wagner HD 1998 The material bone: structure-mechanical function relations. AnRMS 28, 271–298. (doi:10.1146/annurev.matsci.28.1.271)

[2] Buehler MJ 2007 Nano- and micromechanical properties of hierarchical biological materials and tissues. JMatS 42, 8765–8770. (doi:10.1007/s10853-007-1952-8)

[3] Holstein JH, Garcia P, Histing T, Klein M, Becker SC, Menger MD, Pohlemann T 2011 Mouse models for the study of fracture healing and bone regeneration, pp. 175–191. London, UK: Springer.

[4] Elefteriou F, Yang X 2011 Genetic mouse models for bone studies--strengths and limitations. Bone 49, 1242–1254. (doi:10.1016/j.bone.2011.08.021) 21907838 10.1016/j.bone.2011.08.021 PMC3331798

[5] Oyen ML 2010 Handbook of nanoindentation: with biological applications. Singapore: Pan Stanford Publishing.

[6] Vashishth D, Tanner KE, Bonfield W 2001 Fatigue of cortical bone under combined axial-torsional loading. J. Orthop. Res. 19, 414–420. (doi:10.1016/S0736-0266(00)00036-X) 11398854 10.1016/S0736-0266(00)00036-X

[7] Einhorn TA 1992 Bone strength: the bottom line. Calcif. Tissue Int. 51, 333–339. (doi:10.1007/BF00316875) 1458335 10.1007/BF00316875

[8] O'Brien FJ, Hardiman DA, Hazenberg JG, Mercy MV, Mohsin S, Taylor D, Lee TC 2005 The behaviour of microcracks in compact bone. Eur. J. Morphol. 42, 71–79. (doi:10.1080/09243860500096131) 16123026 10.1080/09243860500096131

[9] De Souza RL, Matsuura M, Eckstein F, Rawlinson SC, Lanyon LE, Pitsillides AA 2005 Non-invasive axial loading of mouse tibiae increases cortical bone formation and modifies trabecular organization: a new model to study cortical and cancellous compartments in a single loaded element. Bone 37, 810–818. (doi:10.1016/j.bone.2005.07.022) 16198164 10.1016/j.bone.2005.07.022

[10] Voide R, Schneider P, Stauber M, Wyss P, Stampanoni M, Sennhauser U, Van Lenthe G, Müller R 2009 Time-lapsed assessment of microcrack initiation and propagation in murine cortical bone at submicrometer resolution. Bone 45, 164–173. (doi:10.1016/j.bone.2009.04.248) 19410668 10.1016/j.bone.2009.04.248

[11] Rho JY, Tsui TY, Pharr GM 1997 Elastic properties of human cortical and trabecular lamellar bone measured by nanoindentation. Biomaterials 18, 1325–1330. (doi:10.1016/S0142-9612(97)00073-2) 9363331 10.1016/s0142-9612(97)00073-2

[12] Ferguson VL, Olesiak SE 2010 Nanoindentation of bone, pp 185–237. Singapore: Pan Stanford Publishing.

[13] Dong XN, Guo XE 2004 The dependence of transversely isotropic elasticity of human femoral cortical bone on porosity. J. Biomech. 37, 1281–1287. (doi:10.1016/j.jbiomech.2003.12.011) 15212934 10.1016/j.jbiomech.2003.12.011

[14] Fan Z, Swadener JG, Rho JY, Roy ME, Pharr GM 2002 Anisotropic properties of human tibial cortical bone as measured by nanoindentation. J. Orthop. Res. 20, 806–810. (doi:10.1016/S0736-0266(01)00186-3) 12168671 10.1016/S0736-0266(01)00186-3

[15] Swadener JG, Rho JY, Pharr GM 2001 Effects of anisotropy on elastic moduli measured by nanoindentation in human tibial cortical bone. J. Biomed. Mater. Res. 57, 108–112. (doi:10.1002/1097-4636(200110)57:1<108::AID-JBM1148>3.0.CO;2-6) 11416856 10.1002/1097-4636(200110)57:1<108::aid-jbm1148>3.0.co;2-6

[16] Rho JY, Currey JD, Zioupos P, Pharr GM 2001 The anisotropic Young's modulus of equine secondary osteones and interstitial bone determined by nanoindentation. J. Exp. Biol. 204, 1775–1781. 11316498 10.1242/jeb.204.10.1775

[17] Rho J-Y, Roy ME, Tsui TY, Pharr GM 1999 Elastic properties of microstructural components of human bone tissue as measured by nanoindentation. J. Biomed. Mater. Res. 45, 48–54. (doi:10.1002/(SICI)1097-4636(199904)45:1<48::AID-JBM7>3.0.CO;2-5) 10397957 10.1002/(sici)1097-4636(199904)45:1<48::aid-jbm7>3.0.co;2-5

[18] Carnelli D, Lucchini R, Ponzoni M, Contro R, Vena P 2011 Nanoindentation testing and finite element simulations of cortical bone allowing for anisotropic elastic and inelastic mechanical response. J. Biomech. 44, 1852–1858. (doi:10.1016/j.jbiomech.2011.04.020) 21570077 10.1016/j.jbiomech.2011.04.020

[19] Iyo T, Maki Y, Sasaki N, Nakata M 2004 Anisotropic viscoelastic properties of cortical bone. J. Biomech. 37, 1433–1437. (doi:10.1016/j.jbiomech.2003.12.023) 15275852 10.1016/j.jbiomech.2003.12.023

[20] Carnelli D, Vena P, Dao M, Ortiz C, Contro R 2013 Orientation and size-dependent mechanical modulation within individual secondary osteons in cortical bone tissue. J. R. Soc. Interface 10, 20120953 (doi:10.1098/rsif.2012.0953) 23389895 10.1098/rsif.2012.0953 PMC3627101

[21] Lucchini R, Carnelli D, Ponzoni M, Bertarelli E, Gastaldi D, Vena P 2011 Role of damage mechanics in nanoindentation of lamellar bone at multiple sizes: experiments and numerical modeling. J. Mech. Behav. Biomed. Mater. 4, 1852–1863. (doi:10.1016/j.jmbbm.2011.06.002) 22098884 10.1016/j.jmbbm.2011.06.002

[22] Leong PL, Morgan EF 2008 Measurement of fracture callus material properties via nanoindentation. Acta Biomater. 4, 1569–1575. (doi:10.1016/j.actbio.2008.02.030) 18400573 10.1016/j.actbio.2008.02.030 PMC2575108

[23] Leong PL, Morgan EF 2009 Correlations between indentation modulus and mineral density in bone-fracture calluses. Integr. Comp. Biol. 49, 59–68. (doi:10.1093/icb/icp024) 21669846 10.1093/icb/icp024 PMC3202910

[24] Hoerth RM, Seidt BM, Shah M, Schwarz C, Willie BM, Duda GN, Fratzl P, Wagermaier W 2014 Mechanical and structural properties of bone in non-critical and critical healing in rat. Acta Biomater. 10, 4009–4019. (doi:10.1016/j.actbio.2014.06.003) 24929204 10.1016/j.actbio.2014.06.003

[25] Fischer-Cripps AC 2011 Nanoindentation. New York, NY: Springer.

[26] Oliver WC, Pharr GM 2004 Measurement of hardness and elastic modulus by instrumented indentation: advances in understanding and refinements to methodology. J. Mater. Res. 19, 3–20. (doi:10.1557/jmr.2004.19.1.3)

[27] Fan ZF, Rho JY 2003 Effects of viscoelasticity and time-dependent plasticity on nanoindentation measurements of human cortical bone. J. Biomed. Mater. Res. A 67A, 208–214. (doi:10.1002/jbm.a.10027) 10.1002/jbm.a.10027 14517878

[28] Oliver WC, Pharr GM 2011 An improved technique for determining hardness and elastic modulus using load and displacement sensing indentation experiments. J. Mater. Res. 7, 1564–1583. (doi:10.1557/JMR.1992.1564)

[29] Akhter MP, Fan Z, Rho JY 2004 Bone intrinsic material properties in three inbred mouse strains. Calcif. Tissue Int. 75, 416–420. (doi:10.1007/s00223-004-0241-7) 15592798 10.1007/s00223-004-0241-7

[30] Pathak S, Swadener JG, Kalidindi SR, Courtland HW, Jepsen KJ, Goldman HM 2011 Measuring the dynamic mechanical response of hydrated mouse bone by nanoindentation. J. Mech. Behav. Biomed. Mater. 4, 34–43. (doi:10.1016/j.jmbbm.2010.09.002) 21094478 10.1016/j.jmbbm.2010.09.002 PMC3279917

[31] Rodriguez-Florez N, Oyen ML, Shefelbine SJ 2013 Insight into differences in nanoindentation properties of bone. J. Mech. Behav. Biomed. Mater. 18, 90–99. (doi:10.1016/j.jmbbm.2012.11.005) 23262307 10.1016/j.jmbbm.2012.11.005

[32] Silva MJ, Brodt MD, Fan Z, Rho JY 2004 Nanoindentation and whole-bone bending estimates of material properties in bones from the senescence accelerated mouse SAMP6. J. Biomech. 37, 1639–1646. (doi:10.1016/j.jbiomech.2004.02.018) 15388305 10.1016/j.jbiomech.2004.02.018

[33] Rho JY, Pharr GM 1999 Effects of drying on the mechanical properties of bovine femur measured by nanoindentation. J. Mater. Sci. Mater. Med. 10, 485–488. (doi:10.1023/A:1008901109705) 15348117 10.1023/a:1008901109705

[34] Hofmann T, Heyroth F, Meinhard H, Franzel W, Raum K 2006 Assessment of composition and anisotropic elastic properties of secondary osteon lamellae. J. Biomech. 39, 2282–2294. (doi:10.1016/j.jbiomech.2005.07.009) 16144702 10.1016/j.jbiomech.2005.07.009

[35] Hoffler CE, Guo XE, Zysset PK, Goldstein SA 2005 An application of nanoindentation technique to measure bone tissue Lamellae properties. J. Biomech. Eng. 127, 1046–1053. (doi:10.1115/1.2073671) 16502646 10.1115/1.2073671

[36] Zysset PK, Guo XE, Hoffler CE, Moore KE, Goldstein SA 1999 Elastic modulus and hardness of cortical and trabecular bone lamellae measured by nanoindentation in the human femur. J. Biomech. 32, 1005–1012. (doi:10.1016/S0021-9290(99)00111-6) 10476838 10.1016/s0021-9290(99)00111-6

[37] Hoc T, Henry L, Verdier M, Aubry D, Sedel L, Meunier A 2006 Effect of microstructure on the mechanical properties of Haversian cortical bone. Bone 38, 466–474. (doi:10.1016/j.bone.2005.09.017) 16332459 10.1016/j.bone.2005.09.017

[38] Bushby AJ, Ferguson VL, Boyde A 2011 Nanoindentation of bone: comparison of specimens tested in liquid and embedded in polymethylmethacrylate. J. Mater. Res. 19, 249–259. (doi:10.1557/jmr.2004.19.1.249)

[39] Gupta HS, Stachewicz U, Wagermaier W, Roschger P, Wagner HD, Fratzl P 2011 Mechanical modulation at the lamellar level in osteonal bone. J. Mater. Res. 21, 1913–1921. (doi:10.1557/jmr.2006.0234)

[40] Currey JD 1969 The relationship between the stiffness and the mineral content of bone. J. Biomech. 2, 477–480. (doi:10.1016/0021-9290(69)90023-2) 16335147 10.1016/0021-9290(69)90023-2

[41] Seto J, Gupta HS, Zaslansky P, Wagner HD, Fratzl P 2008 Tough lessons from bone: extreme mechanical anisotropy at the mesoscale. Adv. Funct. Mater. 18, 1905–1911. (doi:10.1002/adfm.200800214)

[42] Francillon-Vieillot H, De Buffrénil V, Castanet JD, Géraudie J, Meunier F, Sire J, Zylberberg L, De Ricqlès A 1990 Microstructure and mineralization of vertebrate skeletal tissues. In Skeletal biomineralization: patterns, processes and evolutionary trends (ed. JG Carter), pp. 175–234. New York, NY: Springer.

[43] Franzoso G, Zysset PK 2009 Elastic anisotropy of human cortical bone secondary osteons measured by nanoindentation. J. Biomech. Eng. 131, 021001 (doi:10.1115/1.3005162) 19102560 10.1115/1.3005162

[44] Wang XJ, Chen XB, Hodgson PD, Wen CE 2006 Elastic modulus and hardness of cortical and trabecular bovine bone measured by nanoindentation. Trans. Nonferrous Metals Soc. China 16, s744–s748. (doi:10.1016/S1003-6326(06)60293-8)

[45] Turner CH, Rho J, Takano Y, Tsui TY, Pharr GM 1999 The elastic properties of trabecular and cortical bone tissues are similar: results from two microscopic measurement techniques. J. Biomech. 32, 437–441. (doi:10.1016/S0021-9290(98)00177-8) 10213035 10.1016/s0021-9290(98)00177-8

[46] Mercer C, He MY, Wang R, Evans AG 2006 Mechanisms governing the inelastic deformation of cortical bone and application to trabecular bone. Acta Biomater. 2, 59–68. (doi:10.1016/j.actbio.2005.08.004) 16701859 10.1016/j.actbio.2005.08.004

[47] Carden A, Rajachar RM, Morris MD, Kohn DH 2003 Ultrastructural changes accompanying the mechanical deformation of bone tissue: a Raman imaging study. Calcif. Tissue Int. 72, 166–175. (doi:10.1007/s00223-002-1039-0) 12469250 10.1007/s00223-002-1039-0

[48] Fantner GE et al. 2005 Sacrificial bonds and hidden length dissipate energy as mineralized fibrils separate during bone fracture. Nat. Mater. 4, 612–616. (doi:10.1038/nmat1428) 16025123 10.1038/nmat1428

[49] Shahar R, Zaslansky P, Barak M, Friesem AA, Currey JD, Weiner S 2007 Anisotropic Poisson's ratio and compression modulus of cortical bone determined by speckle interferometry. J. Biomech. 40, 252–264. (doi:10.1016/j.jbiomech.2006.01.021) 16563402 10.1016/j.jbiomech.2006.01.021

[50] Fratzl P, Gupta HS, Paschalis EP, Roschger P 2004 Structure and mechanical quality of the collagen–mineral nano-composite in bone. J. Mater. Chem. 14, 2115–2123. (doi:10.1039/B402005G)

[51] Dunlop JW, Fratzl P 2013 Multilevel architectures in natural materials. Scr. Mater. 68, 8–12. (doi:10.1016/j.scriptamat.2012.05.045)

[52] Lipson SF, Katz JL 1984 The relationship between elastic properties and microstructure of bovine cortical bone. J. Biomech. 17, 231–240. (doi:10.1016/0021-9290(84)90134-9) 6736060 10.1016/0021-9290(84)90134-9

[53] Yang R, Zhang T, Jiang P, Bai Y 2008 Experimental verification and theoretical analysis of the relationships between hardness, elastic modulus, and the work of indentation. Appl. Phys. Lett. 92, 231906 (doi:10.1063/1.2944138)

[54] Casanova M, Balmelli A, Camelli D, Courty D, Schneider P, Müller R 2017 Data from: Nanoindentation analysis of the micromechanical anisotropy in mouse cortical bone. Dryad Digital Repostory. (http://dx.doi.org/10.5061/dryad.h5p79) 10.1098/rsos.160971 PMC5367284 28386450

