# Supplementary material for: Nanoindentation analysis of the micromechanical anisotropy in mouse cortical bone
Source: R Soc Open Sci. 2017 Feb 22;4(2):160971. doi: 10.1098/rsos.160971 (PMC5367284; doi:10.1098/rsos.160971)
Supplement: Table S1 [file rsos160971supp1.pdf]

Table S1 Summary of the mean values and standard deviations of reduced elastic modulus and hardness found in this study together with their anisotropy ratios.

		Proximal	Central	Distal
		N=18	*N=18*	*N=18*
E_r (GPa)	Transverse	11.8 (3.3)	12.6 (3.2)	12.4 (2.2)
	Longitudinal	19.4 (3.0)***	20.1 (1.9)***	16.4 (3.0)***,# #
	Anisotropy ratio	1.72 (0.40)	1.75 (0.69)	1.34 (0.30)
H (GPa)	Transverse	0.53 (0.09)	0.53 (0.10)	0.52 (0.06)
	Longitudinal	0.71 (0.09)***	0.68 (0.08)***	0.61 (0.10)**, ++
	Anisotropy ratio	1.35 (0.27)	1.35 (0.47)	1.17 (0.19)

***: $p < 0.001$ when compared to transverse values on the same region; **: $p < 0.01$ when compared to transverse values on the same region, ##: $p < 0.01$ when compared to longitudinal values in proximal region and also to longitudinal values in central region; ++: $p < 0.01$ when compared to longitudinal values in proximal region.
