# Supplementary material for: Nanoindentation analysis of the micromechanical anisotropy in mouse cortical bone
Source: R Soc Open Sci. 2017 Feb 22;4(2):160971. doi: 10.1098/rsos.160971 (PMC5367284; doi:10.1098/rsos.160971)

Figure S1 Values of reduced modulus (E_r) and hardness (H) for each set of indentations in both transverse (left) and longitudinal (right) directions. P: proximal region; C: central region; D: distal region. Mxx: mouse number and right or left femur (e.g. M1L: mouse no. 1, left femur).

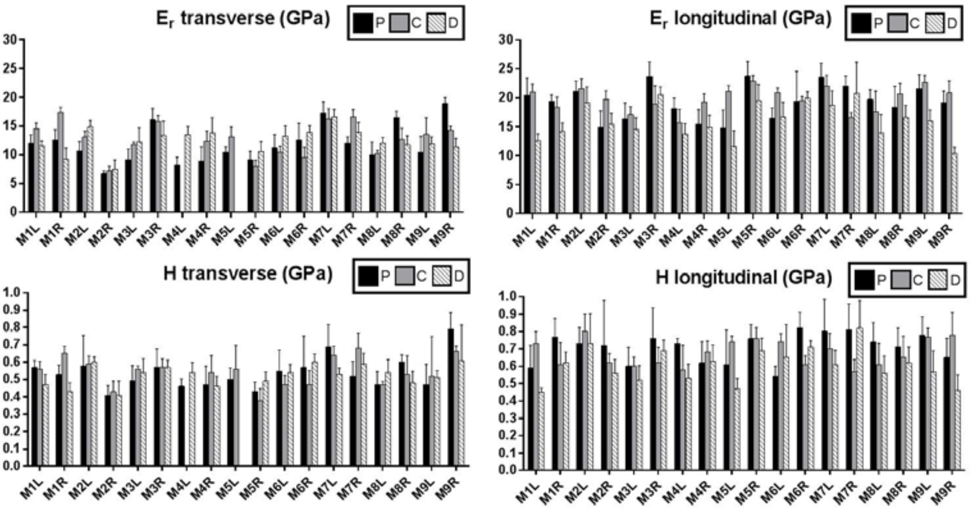

Supplement: Figure S1 [file rsos160971supp2.pdf]
